# Anisogamy explains why males benefit more from additional matings

**DOI:** 10.1038/s41467-022-31620-w

**Published:** 2022-07-06

**Authors:** Jonathan M. Henshaw, Adam G. Jones, Lukas Schärer

**Affiliations:** 1 Institute of Biology I, University of Freiburg, Hauptstraße 1, D-79104 Freiburg, Germany; 2 Department of Biological Sciences, University of Idaho, 875 Perimeter Drive, Moscow, ID 83844 USA; 3 Zoological Institute, Department of Environmental Sciences, University of Basel, CH-4051 Basel, Switzerland

**Keywords:** Evolutionary theory, Sexual selection, Evolutionary ecology, Social evolution

## Abstract

Why do males typically compete more intensely for mating opportunities than do females and how does this relate to sex differences in gamete size? A new study provides a formal evolutionary link between gamete size dimorphism and ‘Bateman gradients’, which describe how much individuals of each sex benefit from additional matings.

Male and female animals typically differ in their behaviour, particularly when it comes to competing for mates and provisioning offspring. Despite decades of empirical and theoretical research, the evolutionary origins of such sex differences remain contentious. One reason for this controversy is obvious: our perception of sex differences in humans inevitably influences how we see animals^1^. A more fundamental reason, however, is that the patchwork quilt of animal sex differences is complex and richly patterned in a way that defies simple explanations^2,3^. Males and females differ in astoundingly diverse ways across species; and yet, some kinds of differences are far more common than others^4^. Here are two closely linked examples: First, males typically, but by no means always, compete more intensely for access to mating partners than do females^5^. Second, a male’s reproductive success tends to increase more steeply with its number of mating partners^6^. The slope of this relationship is known as the ‘Bateman gradient’ after geneticist John Angus Bateman, who first drew attention to this widespread sex difference and its evolutionary importance (Fig. 1)^7,8^. Steeper Bateman gradients reflect a stronger fitness incentive for individuals to seek out or compete for additional mating opportunities. Although these gradients are typically steeper in males, the reverse pattern can occur in taxa with intense mating competition among females, such as in the pipefish family Syngnathidae^6^. Bateman gradients have proved useful as both a conceptual tool for understanding mating system evolution and a summary statistic of the empirical intensity of mating competition in a given sex and species.

**Fig. 1:**
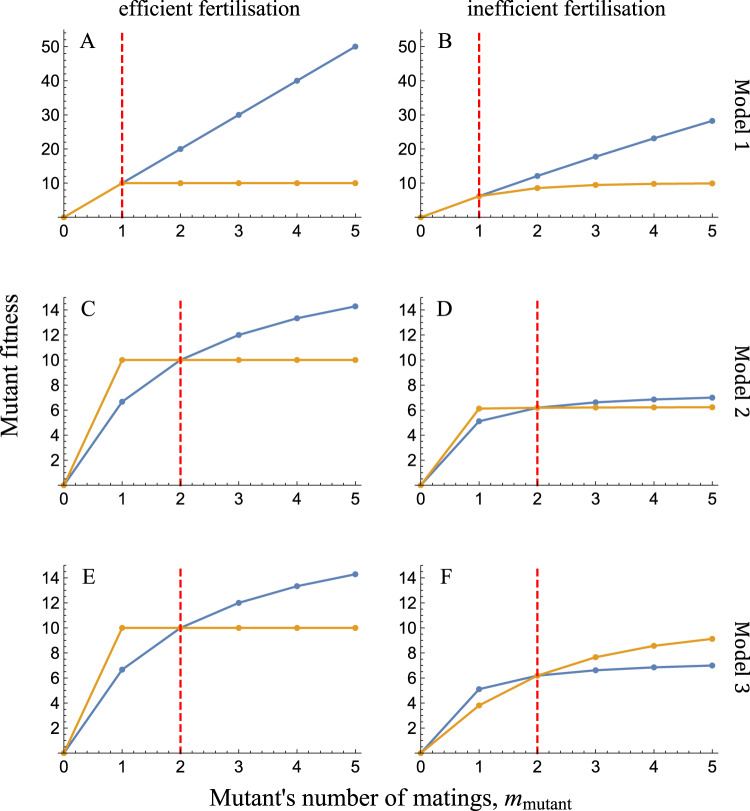
Bateman gradients of mutant males (blue) and females (yellow) in the three models of Lehtonen^12^. The structure of each model is outlined in Fig. 2. Fertilisation is either efficient (**A**, **C**, **E**) or inefficient (**B**, **D**, **F**). The resident number of matings is indicated by the dashed red lines. Under most circumstances, a mutant male’s fitness increases more steeply than a mutant female’s fitness with its number of matings (**A**–**E**). However, under inefficient internal fertilisation with a low anisogamy ratio (i.e. few sperm for each egg), Bateman gradients can theoretically reverse, so that female fitness increases more steeply with the number of matings (**F**). In this illustration, females produce ten eggs each and males produce one hundred sperm. Resident individuals are monogamous (Model 1), participate in two spawning groups (Model 2), or mate twice (Model 3). Fertilisation efficiency is given by *a* = 1 (‘efficient fertilisation’) or *a* = 0.01 (‘inefficient fertilisation’) (see ref. ^12^ for parameter definitions).

Attempts to explain the evolutionary origins of sex differences in simple terms are sometimes seen as reductionist and—in the most literal sense of the word—they are. But while abstract models will never explain the rich diversity of sex differences seen in nature, they can be invaluable in pinpointing the key variables and revealing the broader picture. Several recent models have linked anisogamy—the dimorphism in gamete size that is the definitional difference between male and female—to the evolution of secondary sex differences in parental care and mating competition^9–11^. In a recent article in *Nature Communications*, Lehtonen makes an elegant contribution by establishing a formal link between anisogamy and Bateman gradients for the first time^12^. Lehtonen’s models formally support Bateman’s assertion that “the primary cause of intra-masculine selection would thus seem to be that females produce much fewer gametes than males”^7^.

## From anisogamy to the Bateman gradient

**Fig. 2:**
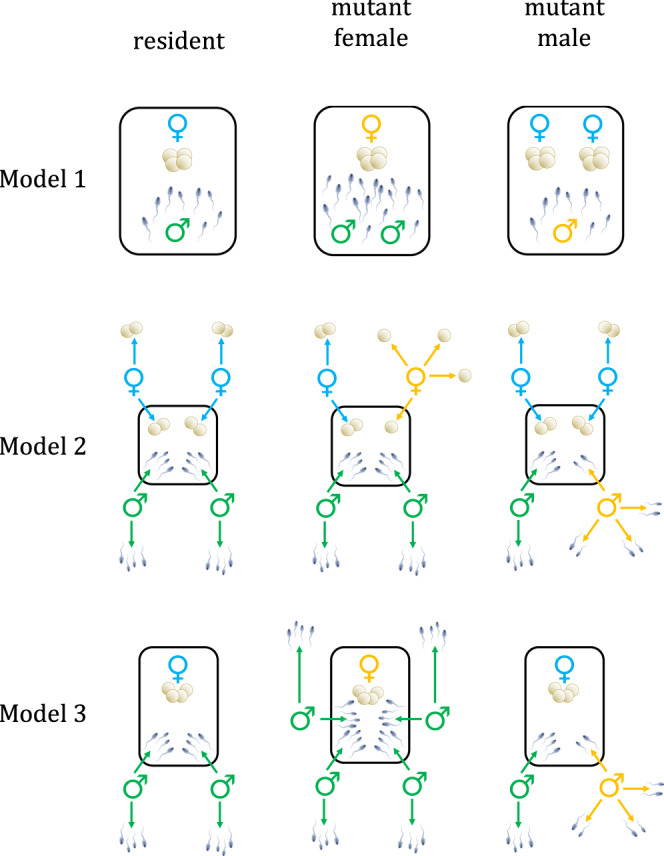
Structure of the three models of Lehtonen^12^, showing differences in mating behaviour between resident males (green), resident females (blue) and mutant males and females (both yellow). For illustration, we suppose that females produce four eggs each and males produce eight sperm (the anisogamy ratio in nature is typically much higher). In Model 1, resident individuals spawn monogamously in a ‘nest’ (black outline), whereas mutant males and females can bring additional partners to their nest to spawn in a group. In Model 2, resident individuals divide their gametes equally among *m* spawning groups, each consisting of *m* individuals of each sex (shown here with *m* = 2). Mutant males and females instead divide their gametes among a larger or smaller number of groups, *m*_mutant_ (shown here with *m*_mutant_ = 4). In Model 3, there is a further sex asymmetry in addition to anisogamy: Fertilisation takes place inside the female’s body. Resident individuals mate with *m* partners (shown here with *m* = 2), whereas mutant males and females mate with a larger or smaller number of partners, *m*_mutant_ (shown here with *m*_mutant_ = 4).

Lehtonen^12^ presents three simple models with the same broad structure: a single mutant individual with divergent mating behaviour arises in a population of ‘residents’ that all play the same strategy, and the success of that mutant is then followed (Figs. 1, 2). Specifically, Lehtonen investigates the fitness benefits of increased mating for mutant males in comparison to mutant females. Two important parameters can be varied: (i) the degree of anisogamy (defined here as the ratio of sperm number to egg number), which captures how divergent males and females are in the size (and thus number) of gametes they produce, and (ii) the efficiency of fertilisation, which determines how easily gametes can find and fuse with each other. If fertilisation is highly efficient, then gametes of the less numerous type will achieve nearly full fertilisation; on the other hand, inefficient fertilisation can result in gametes of both sexes going unfertilised.

In the first two models, fertilisation is external and no assumptions are made about pre-existing differences between the sexes apart from the number of gametes they produce. In other words, males and females are identical except that males produce sperm in greater numbers than females produce eggs. In Model 1, resident individuals are assumed to mate monogamously, whereas a mutant can monopolise multiple partners of the opposite sex (Fig. 2). Importantly, both male and female mutants can bring additional partners back to their ‘nest’ to spawn in a group. When fertilisation is highly efficient, females can fertilise all of their eggs by bringing back a single male, and there is simply no benefit (in this model) of seeking further partners (Fig. 1A). In contrast, anisogamy means that males always produce at least some gametes in excess, and thus can benefit from seeking additional mates. When fertilisation is inefficient, however, both sexes benefit from increasing the concentration of opposite-sex gametes at their ‘nest’ (Fig. 1B). This latter benefit is sex-symmetric, whereas the former continues to apply only to males. As a consequence, the Bateman gradients are always steeper for males than for females (Fig. 1A, B), confirming Bateman’s argument.

Model 2 similarly assumes external fertilisation, but in this case the resident males and females meet in groups consisting of *m* individuals of each sex (Fig. 2). Fertilisation occurs via group spawning. It is assumed that each resident individual divides its gametes evenly across *M* groups, whereas mutant individuals can instead spread their gametes over a larger or smaller number of groups (note that the author assumes that *M* = *m*, but this assumption could be relaxed without undermining the core argument). Spreading gametes out across a larger number of spawning groups does not increase the concentration of opposite-sex gametes they encounter (Fig. 2). However, a mutant that spreads its gametes more widely reduces the density of its own gametes across those groups in which it spawns. This in turn results in there being more opposite-sex gametes for each gamete of the mutant’s sex in those groups. For example, in Fig. 2, mutant males spawn in twice as many groups as resident males and thereby halve the density of their own sperm in each group. The resulting egg-to-sperm ratio of $\frac{4}{6}=\frac{2}{3}$ is more favourable than the ratio of $\frac{4}{8}=\frac{1}{2}$ that the resident males experience. Mutant females can similarly increase local sperm-to-egg ratios by spreading their eggs over more groups. However, in contrast to males, this only leads to fitness benefit if fertilisation is inefficient, and even then the benefit to females is very modest (scarcely perceptible in Fig. 1D). Gamete spreading reduces wasteful competition among the mutants’ own gametes for fertilisation. Such ‘local’ gamete competition, like gamete competition more generally, is stronger among sperm than among eggs because sperm are more numerous under anisogamy^13,14^. Consequently, as in Model 1, Bateman gradients are always steeper in males (Fig. 1C, D). Recall that the results of the above models emerge in the absence of any assumptions beyond the sex difference in the number of gametes produced.

The third and final model allows for a further pre-existing difference between the sexes in addition to anisogamy: internal fertilisation, which is common and widespread in animals (Fig. 2)^15^. Each female is assumed to mate with *m* males, while each male divides his gametes evenly among *m* females. As in the previous two models, males benefit more than females from additional matings under most conditions. However, in the particular case where fertilisation is highly inefficient and the ratio of sperm to eggs is not too large, the pattern can theoretically reverse, such that female Bateman gradients exceed their male counterparts (Fig. 1F). The reason is that the effects of gamete concentration are asymmetric under internal fertilisation: Multiple mating by a female increases the local concentration of sperm its eggs experience, whereas a male’s multiple mating does not increase the concentration of eggs around its sperm (Fig. 2). Under conditions of severe sperm limitation—due to both weak anisogamy and highly inefficient fertilisation—this can lead to females benefitting more from additional matings than males (Fig. 1F). Although intriguing, it is unclear whether this finding has any empirical relevance, as sperm limitation is probably rarely severe in internal fertilisers. Under more realistic conditions of moderate to high fertilisation rates, sex differences in the degree of local gamete competition once again become decisive, and male Bateman gradients exceed their female counterparts (Fig. 1E).

## What can we learn from a simple model?

The models of Lehtonen^12^ provide a series of instructive ‘base cases’ for understanding why Bateman gradients are typically higher in males. Importantly, by assuming that male fitness is limited only by fertilisations, they implicitly assume that males do not contribute to provisioning offspring, except possibly via their gametes in weakly anisogamous species. This reflects the empirical situation in a large majority of species, including both animals and other anisogamous groups such as multicellular plants. These models consequently help to explain the dominant patterns of mating competition in nature—an important goal—but do not fully explain why Bateman gradients are reversed in some animal species^6^. A great strength of these models is their disarming simplicity, which allows readers to follow the logic without the need to reflect on complex mathematics.

A few caveats should be kept in mind in interpreting the models. First, the Bateman gradients imagined by Lehtonen differ somewhat from those measured under natural conditions^6,16^. When measured in freely mating populations, Bateman gradients reflect both the benefits and the costs of mating multiply. In contrast, the Bateman gradients in the models here are closer in spirit to an experiment in which mutant individuals are provided with additional mates by the experimenter in a way that is cost-free^16^.

Further, the models explore no evolutionary feedbacks from the sex differences in Bateman gradients to mating behaviour and patterns at the population level, which in turn are bound to shape the Bateman gradients under natural conditions. If seeking or competing for additional mating partners is costly under natural conditions, then males—being the sex with more to gain from additional matings—will typically be more willing to accept such costs. Moreover, due to local sperm competition, a reduction in sperm production is often less costly in fitness terms than a proportional reduction in eggs^13^. This effect is simple to demonstrate in the models of Lehtonen, although the author does not emphasise it. Rediverting resources away from gamete production and towards alternative fitness routes is consequently more often beneficial for males than females^9^. An interesting case that is worthy of greater theoretical attention is the redirection of resources towards paternal care, which can feed back to reduce male Bateman gradients.

These new models of the evolutionary link between anisogamy and Bateman gradients are very much in the spirit of Robert Trivers’ famous thought experiments^8^. We expect that their elegance and accessibility will inspire and inform the debate on the evolutionary origins of sex differences.
